# First report on detection of *Hepatozoon ayorgbor* in *Rhipicephalus haemaphysaloides* and *Hepatozoon colubri* in *Haemaphysalis sulcata* and *Hyalomma anatolicum*: risks of spillover of *Hepatozoon* spp. from wildlife to domestic animals

**DOI:** 10.3389/fvets.2023.1255482

**Published:** 2023-09-18

**Authors:** Hadia Tila, Mehran Khan, Mashal M. Almutairi, Abdulaziz Alouffi, Haroon Ahmed, Tetsuya Tanaka, Kun-Hsien Tsai, Abid Ali

**Affiliations:** ^1^Department of Zoology, Abdul Wali Khan University Mardan, Mardan, Khyber Pakhtunkhwa, Pakistan; ^2^Department of Pharmacology and Toxicology, College of Pharmacy, King Saud University, Riyadh, Saudi Arabia; ^3^Department of Biotechnology, King Abdulaziz City for Science and Technology, Riyadh, Saudi Arabia; ^4^Department of Biosciences, COMSATS University Islamabad (CUI), Islamabad, Pakistan; ^5^Laboratory of Infectious Diseases, Joint Faculty of Veterinary Medicine, Kagoshima University, Kagoshima, Japan; ^6^Department of Public Health, Institute of Environmental and Occupational Health Sciences, College of Public Health, National Taiwan University, Taipei, Taiwan

**Keywords:** ticks, *Hepatozoon ayorgbor*, *Hepatozoon colubri*, *Hepatozoon canis*, spillover

## Abstract

This study aimed to detect *Hepatozoon* spp. in ticks infesting asymptomatic domestic animals and to provide insight into their potential spillover from wild to domestic animals. In total, 537 tick specimens were collected in Khyber Pakhtunkhwa, Pakistan, and morphologically identified. The most prevalent tick species was *Haemaphysalis cornupunctata* (69; 12.8%), followed by *Haemaphysalis kashmirensis* (62; 11.5%), *Rhipicephalus microplus* (58; 10.8%), *Haemaphysalis montgomeryi* (51; 9.5%), *Rhipicephalus sanguineus* (49; 9.1%), each *Haemaphysalis bispinosa* and *Haemaphysalis sulcata* (43; 8.0%), each *Hyalomma anatolicum* and *Rhipicephalus turanicus* (37; 6.9%), *Rhipicephalus haemaphysaloides* (33; 6.1%) *Hyalomma scupense* (30; 5.6%), and *Hyalomma isaaci* (25; 4.7%). The extracted DNA from a subset of each tick species was subjected to PCR to amplify 18S rRNA fragments of *Hepatozoon* spp. By BLAST analysis, the *Hepatozoon* sp. detected in *Hy. anatolicum* infesting cows and in *Ha. sulcata* infesting sheep showed 99.7% maximum identity with *Hepatozoon colubri*. Similarly, the *Hepatozoon* sp. detected in *R. haemaphysaloides* infesting goats shared 99.49% maximum identity with *Hepatozoon ayorgbor*, and the *Hepatozoon* sp. detected in *R. sanguineus* infesting dogs exhibited 99.7% identity with *Hepatozoon canis*. Having an overall infection rate (9.3%; 16/172), the highest infection rate was recorded for each *H. canis*, and *H. colubri* (3.5%; 6/172), followed by *H. ayorgbor* (2.3%; 4/172). In the phylogenetic tree, *H. colubri* clustered with corresponding species from Iran, *H. ayorgbor* clustered with the same species from Croatia, Ghana, and Portugal, and *H. canis* clustered with the conspecifics from Iran, Israel, Romania, and Zambia. Regarding the potential spillover of *Hepatozoon* spp. from wildlife through ticks, free ranging animals was at higher risk compared to confined animals (RR = 3.05), animals consuming food from wildlife habitats were at higher risk compared to those consuming domestic food (RR = 3.06), and animals residing in farm buildings located in wildlife habitats were at higher risk compared to those residing in farm buildings located in villages (RR = 3.28). In addition to the first report on *H. canis* in *R. sanguineus* in Pakistan, this is the earliest data showing *H. ayorgbor* in *R. haemaphysaloides* and *H. colubri* in *Ha. sulcata* and *Hy. anatolicum*. These preliminary findings suggest a potential spillover of *Hepatozoon* spp. from wild to domestic animals via ticks under certain risk factors.

## Introduction

*Hepatozoon* is a diverse genus of apicomplexan, primarily comprised of haemoparasites transmitted by arthropods, especially by ticks to all classes of terrestrial vertebrates, including wild and domestic animals (1). *Hepatozoon* is comprised of more than 300 apicomplexan haemoparasites, with ~50 species in mammals and 130 species in snakes (1–3). This genus was assigned to Haemogregarinidae prior to allocating to the family Hepatozoidae (4). Due to encompassing some distinct lineages, certain studies have proposed that *Hepatozoon* spp. may not belong to a single genus (5, 6). *Hepatozoon* spp. are globally distributed depending on multiple factors, including the abundance of vertebrate and arthropod hosts (1). With a heteroxenous life cycle, *Hepatozoon* spp. exhibit sporogonic development and oocyst generation occurs in arthropods, while merogony and gametogony in their vertebrate hosts (1, 7, 8).

As intermediate hosts, *Hepatozoon* spp. infect all classes of terrestrial vertebrates, including wild and domestic animals. With low host specificity, depending on the species, some *Hepatozoon* spp. are more prevalent in a particular group of hosts (9). For instance, *Hepatozoon ayorgbor* largely infects wild snakes and wild rodents, whereas *Hepatozoon colubri* mainly infects wild snakes, while *Hepatozoon canis* primarily infects domestic and wild canids. However, *Hepatozoon* spp. can cross-over from their specific wild hosts to unnatural wild and domestic hosts through different agents such as ticks (10–13). In the case of spillover, *Hepatozoon* spp. are considered more pathogenic in unnatural hosts compared to their specific hosts (14–16).

Hematophagous arthropods get infected with *Hepatozoon* spp. by feeding on vertebrate hosts and subsequently act as the definitive host and vectors (17). For instance, mosquitoes serve as principal vectors for *H. ayorgbor* (1, 18), while *Rhipicephalus sanguineus* is the main vector for *H. canis* (1, 19, 20). The principal vectors of many *Hepatozoon* spp., such as *H. colubri*, are not well-established yet. Unlike other tick-borne pathogens which are mainly transmitted during tick bite, *Hepatozoon* spp. are transmitted when vertebrate hosts ingest infected ticks (21, 22). Other transmission routes include via predation of prey, such as *H. ayorgbor*, transmission to snakes through feeding on rodents (18), and vertical transmission such as *H. canis* in *R. sanguineus* (23, 24).

The morphometrics of the peripheral blood gamonts seen in the hosts' blood smears were previously the most widely employed techniques for the identification of *Hepatozoon* spp. However, the limited number of apparent structures and few morphological features make it difficult and unreliable to differentiate *Hepatozoon* spp. (25). A sensitive method for detecting *Hepatozoon* spp. is molecular analysis based on the amplification and sequencing of 18S rRNA fragments (26, 27). Furthermore, ticks have been largely neglected as vectors of *Hepatozoon* spp. and little data is available on *Hepatozoon* spp. of wildlife origin in ticks infesting domestic animals. Therefore, the purpose of this study was to detect *Hepatozoon* spp. in ticks infesting domestic animals and to provide insight into their possible spillover.

## Materials and methods

### Ethical approval

The Advanced Studies and Research Board of Abdul Wali Khan University, Mardan (AWKUM) granted ethical permission (Dir/A&R/AWKUM/2018/1410) for the conduction of this study. Additionally, the owners of the animals also gave their oral permission to collect ticks from their animals.

### Study area

**Figure 1:**
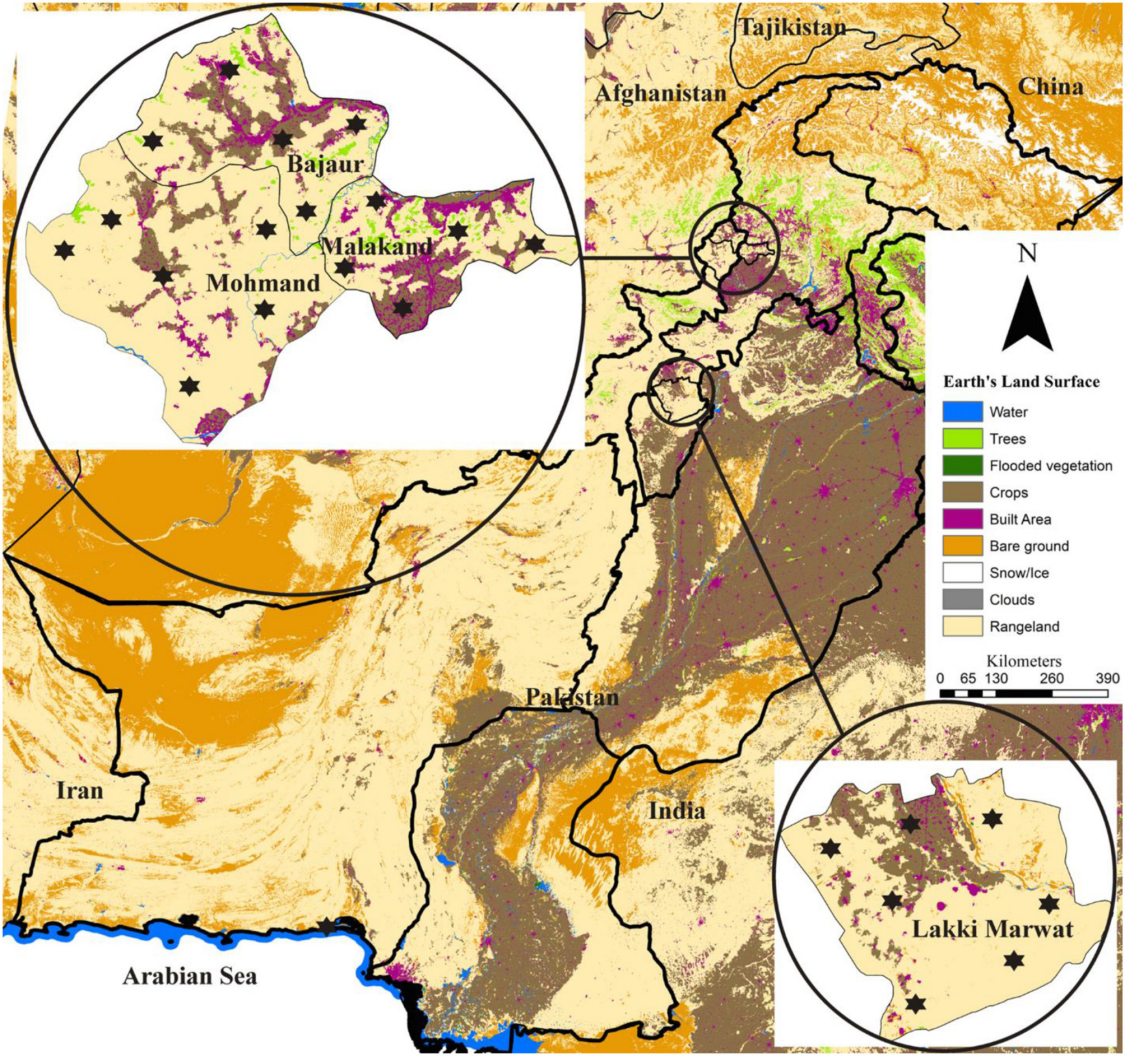
Locations (marked by an asterisk) where domestic hosts were examined for ticks are shown on the land-use and land-cover based map.

This study was conducted in four districts of Khyber Pakhtunkhwa, including Bajaur (34.7865° N, 71.5249° E), Malakand (34.5030° N, 71.9046° E), Mohmand (34.5356° N, 71.2874° E) in the north-western and Lakki Marwat (32.6135° N, 70.9012° E) in the south-eastern. Global Positioning System was used for finding the actual coordinates of collection points in the studied area and subsequently used in designing the map using ArcGIS v 10.3.1 (ESRI, Redlands, CA, USA; Figure 1).

### Tick collection, preservation, and identification

Ticks were randomly collected using forceps from different asymptomatic domestic animals, including cows, livestock guardian dogs, goats, and sheep from March 2022 to September 2022. The appropriate information was recorded during tick collection, including the host type, collection date, and collection site, as well as general observations about the field. Furthermore, the owners of domestic animals were given a questionnaire to complete in order to gather possible risk factors for *Hepatozoon* spp. spillover. The questionnaire consisted of closed-ended questions about host-related details such as gender, age, living status, farming type, food supply, and farm building location, nature, and its altitude. It also contained open-ended questions regarding wildlife and anthropogenic activities such as land use and land cover changes, and human-wildlife conflict. Ticks were stored in a mixture of 95% ethanol, 4% distilled water, and 1% glycerol in properly labeled tubes after cleaning with distilled water followed by 70% ethanol. Using a stereomicroscope (Stemi 508, Zeiss, Germany), tick specimens were morphologically identified based on various morphological features, and validated up to the species level using the standard taxonomic identification keys (28–32).

### DNA extraction and polymerase chain reaction

A sum of 172 tick specimens (one adult female, one male, and two nymphs per species per district) were selected and subjected to DNA extraction individually. Prior to DNA extraction using the phenol-chloroform method with minor modifications (33), the homogenization of ticks was individually carried out using a hygienic scissor coupled with a sterile pestle in a 1.5 ml Eppendorf tube. The extracted DNA pellet was hydrated with 20–30 μl (depending on the tick size) of “nuclease-free” water and subjected to NanoDrop spectrophotometer (Nano-Q, Optizen, South Korea) to measure its quality and quantity.

For the amplification of 18S rRNA fragments of *Hepatozoon* spp., the extracted genomic DNA samples were subjected to conventional PCR (BIOER, China) using specific primers: HEP2 144-169-F (5′-GGTAATTCTAGAGCTAATACATGAGC-3′) and HEP2 743-718-R (5′-ACAATAA AGTA AAAAACAYTTCAAAG-3′), and PCR experimental conditions were set as previously described (34). Each PCR reaction of 25 μL volume contained the following components: 8.5 μL of PCR water “nuclease free,” 1 μL of each primer with a concentration of 10 pmol/μL, 2 μL of extracted DNA (50–100 ng/μL), and 12.5 μL *DreamTaq* green MasterMix (2X). All PCR procedures employed PCR water as a negative control and *H. canis* DNA as a positive control. The obtained amplicons were run on a 2% agarose gel followed by observing via Gel Documentation (BioDoc-It™ Imaging Systems, Upland, CA, USA) and purified using the GeneClean II Kit (Qbiogene, Illkirch, France).

### DNA sequencing and phylogenetic analysis

The purified amplicons were submitted for DNA sequencing (Macrogen, Inc., Seoul, South Korea) by Sanger sequencing method using an ABI 373XL system. Low-quality nucleotide sequences and contaminated reads were eliminated during trimming and assembling of raw sequences through SeqMan version 5.0 (DNASTAR; DNASTAR, Inc., Madison, WI, USA). With the aim to determine the closest identities with other sequences previously deposited in the GenBank, the cleaned sequences were subjected to BLAST (Basic Local Alignment Search Tool) at NCBI (National Center for Biotechnology Information). For the subsequent phylogenetic tree using Molecular Evolutionary Genetics Analysis (MEGA-X) (35), the homologous sequences found in the BLAST results were downloaded in FASTA format. These sequences along with a suitable outgroup were aligned in BioEdit alignment editor v 7.0.5 (36) using ClustalW Multiple alignment (37). The alignment was used for constructing a phylogenetic tree with 1,000 bootstrap replicates under Maximum Likelihood method.

### Statistical analysis

The questionnaire data along with PCR data were collected and assembled in spreadsheets using Excel (Microsoft V, 2016). Using GraphPad Prism version 5.0 (GraphPad Software Inc., San Diego, CA, USA), the assembled data was subjected to chi-square test (χ^2^), selecting significance at *P* < 0.05, and relative risk (RR), with the 95% confidence interval.

## Results

### Tick and host description

Altogether, 537 ticks were morphologically identified, which were belonging to three distinct genera of hard ticks, including *Haemaphysalis, Hyalomma*, and *Rhipicephalus*. Herein, majority of the ticks were *Haemaphysalis cornupunctata* (69, 12.8%), followed by *Haemaphysalis kashmirensis* (62, 11.5%), *Rhipicephalus microplus* (58, 10.8%), *Haemaphysalis montgomeryi* (51, 9.5%), *R*. *sanguineus* (49, 9.1%), each *Haemaphysalis bispinosa* and *Haemaphysalis sulcata* (43, 8.0%), each *Hyalomma anatolicum* and *Rhipicephalus turanicus* (37, 6.9%), *Rhipicephalus haemaphysaloides* (33, 6.1%) *Hyalomma scupense* (30, 5.6%), and *Hyalomma isaaci* (25, 4.7%). The highest prevalence of tick infestation was recorded on dogs (68.4%: 13/19), followed by cows (64.7%: 22/34), goats (58%: 29/50), and sheep (56.3%: 27/48), resulting in overall prevalence (60.9%; 92/151). The details about each tick species, including life stage, host association, and collection site, are provided in Table 1.

**Table 1:** Data about tick species, associated *Hepatozoon* spp., corresponding vertebrate hosts, locality, and molecular analysis.

**Tick species**	**Tick life stages**			**Total ticks**	**Tick hosts**	**Tick collection sites**	**PCR details**		
	**Female**	**Male**	**Nymph**				**Ticks subjected to PCR**	**Positive ticks**	***Hepatozoon*** **spp. detected**
*Ha*. *cornupunctata*	35	20	14	69	Goats, Sheep	Bajaur, Malakand, Mohmand	3F, 3M, 6N	0	
*Ha*. *bispinosa*	17	15	11	43	Goats, Sheep	Bajaur, Malakand, Mohmand	3F, 3M, 6N	0	
*Ha*. *kashmirensis*	28	21	13	62	Goats, Sheep	Bajaur, Malakand, Mohmand	3F, 3M, 6N	0	
*Ha*. *montgomeryi*	22	18	11	51	Goats, Sheep	Bajaur, Malakand, Mohmand	3F, 3M, 6N	0	
*Ha*. *sulcata*	16	15	12	43	Goats, Sheep^*^	Bajaur^*^, Malakand, Mohmand	3F, 3M, 6N	3N	*H*. *colubri*
*Hy*. *anatolicum*	18	9	10	37	Cows^*^, Goats, Sheep	Bajaur^*^, Malakand, Mohmand, Lakki Marwat^*^	4F, 4M, 8N	1F, 2N	*H*. *colubri*
*Hy*. *scupense*	11	10	9	30	Cows	Bajaur, Malakand, Mohmand, Lakki Marwat	4F, 4M, 8N	0	
*Hy*. *isaaci*	10	7	8	25	Cows, Goats, Sheep	Bajaur, Malakand, Mohmand, Lakki Marwat	4F, 4M, 8N	0	
*R*. *haemaphysaloides*	14	10	9	33	Dogs, Goats^*^, Sheep	Bajaur, Malakand^*^, Mohmand^*^, Lakki Marwat	4F, 4M, 8N	1F, 3N	*H*. *ayorgbor*
*R*. *microplus*	22	18	18	58	Cows, Goats, Sheep	Bajaur, Malakand, Mohmand, Lakki Marwat	4F, 4M, 8N	0	
*R*. *sanguineus*	21	15	13	49	Dogs^*^, Goats, Sheep	Bajaur^*^, Malakand^*^, Mohmand, Lakki Marwat^*^	4F, 4M, 8N	2F, 4N	*H*. *canis*
*R*. *turanicus*	15	12	10	37	Dogs, Goats, Sheep	Bajaur, Malakand, Mohmand, Lakki Marwat	4F, 4M, 8N	0	
Total	229	170	138	537			172	16	

^*^Associated with Hepatozoon spp. positive ticks.

### Detection of *Hepatozoon* spp. in ticks

According to BLAST analysis, *Hepatozoon* sp. detected in *Hy. anatolicum* from cows and *Ha. sulcata* from sheep shared a maximum identity of 99.66% with *Hepatozoon colubri*, whereas *Hepatozoon* sp. detected in *R. haemaphysaloides* from goats revealed a maximum identity of 99.49% with *Hepatozoon ayorgbor*, and *Hepatozoon* sp. detected in *R. sanguineus* from dogs showed a maximum identity of 99.66% with *Hepatozoon canis*. With a total infection rate (9.3%, 16/172), the highest rate was recorded for each *H*. *canis*, and *H*. *colubri* (3.5%, 6/172), followed by *H*. *ayorgbor* (2.3%, 4/172). Table 1 provides additional information regarding *Hepatozoon* spp., including the associated vertebrate hosts and the corresponding locality.

The obtained sequences for 18S rRNA were submitted to GenBank under the following accession numbers; OR241141 (*H. ayorgbor*), OR241161 (*H. colubri*), and OR241147 (*H. canis*).

### Sequence and phylogenetic analysis

A sum of 64 sequences (four sequences, two being forward and two reverse per detection) were obtained from all amplified fragments. All sequences of *Hepatozoon* sp. amplified from the genomic DNA of *Hy. anatolicum* and *Ha. sulcata* were identical to each other, resulting in a consensus sequence of 590 bp. Similarly, all sequences of *Hepatozoon* sp. amplified from the genomic DNA of *R. haemaphysaloides* were identical, while all sequences amplified from the genomic DNA of *R. sanguineus* were identical, yielding a consensus sequence of 588 and 596 bp, respectively. The *Ha. sulcata* and *Hy. anatolicum* based sequences showed the highest identity of 99.66% with *H. colubri* (MN723844), while the *R. haemaphysaloides* based sequence displayed the highest identity of 99.49% with *H. ayorgbor* (EF157822.1), as well as with two undetermined *Hepatozoon* spp. (MT919387 and MT919388), and *R. sanguineus* based sequence revealed maximum identity of 99.66% with *H. canis* (KX712126 and KX880505). The details regarding the infection rate and the association of *Hepatozoon* spp. with tick species in each district are shown in Table 1.

**Figure 2:**
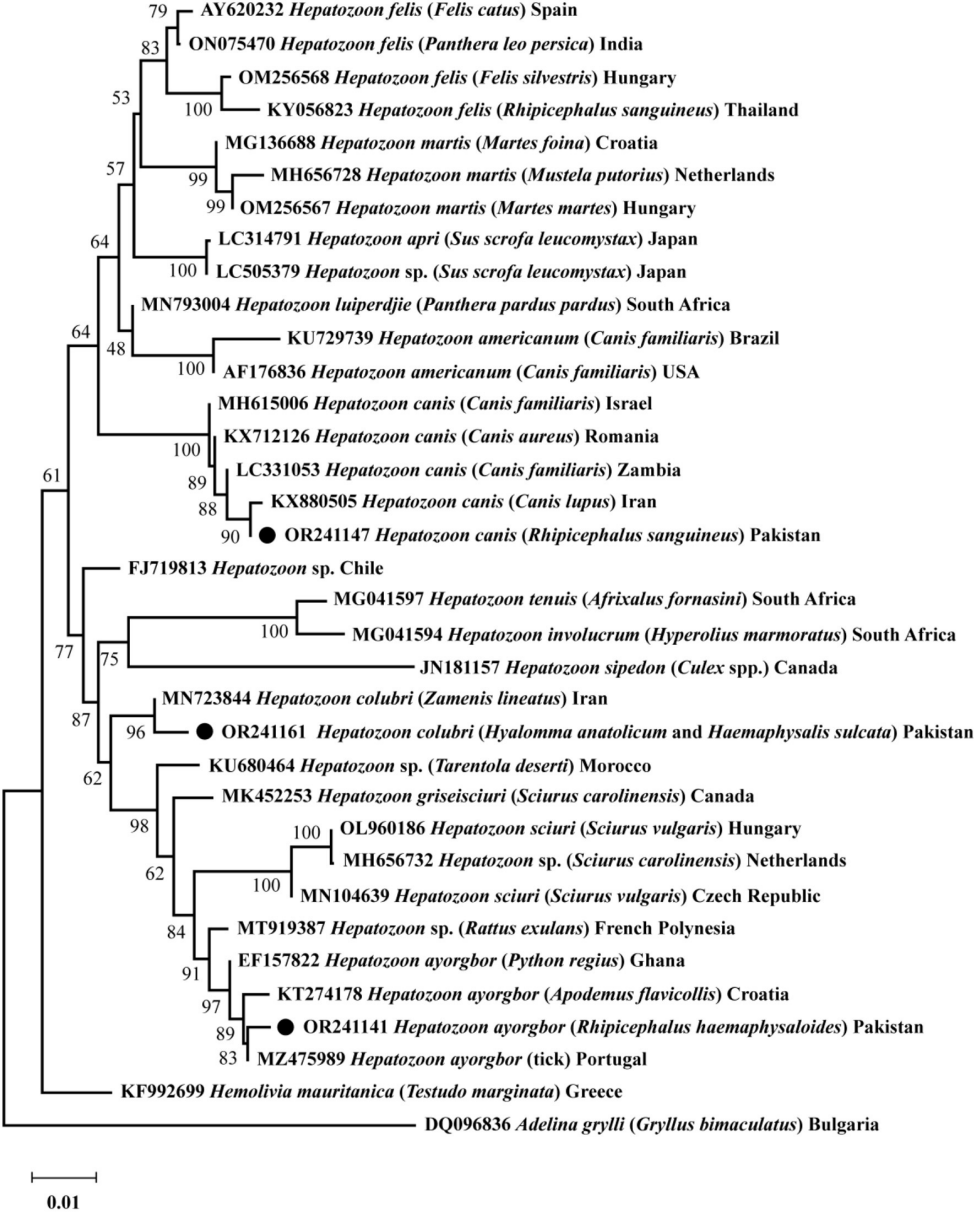
The phylogenetic tree was constructed for *Hepatozoon* spp. based on 18S rRNA sequences. The Neighbor-Joining method was used with 1,000 replicates for bootstrap analysis. The tree was rooted and subrooted using the 18S rRNA sequence of *Adelina grylli* and *Hemolivia mauritanica*, respectively. The GenBank accession numbers, species names, sources, and geographic locations were assigned to all sequences. The *Hepatozoon* spp. detected in this study are marked with a black circle.

Based on 18S rRNA, a phylogenetic tree was obtained encompassing the three *Hepatozoon* spp. detected in the present survey. *Hepatozoon ayorgbor* clustered with the same species previously detected in *Apodemus flavicollis, Python regius*, and an unknown tick species from Croatia, Ghana, and Portugal, respectively. *Hepatozoon colubri* clustered with the corresponding species found in *Zamenis lineatus* from Iran, and *H. canis* clustered with conspecifics detected in *Canis lupus, Canis aureus*, and *Canis familiaris* from Iran, Romania, and Israel and Zambia, respectively (Figure 2).

### Risk factors associated with *Hepatozoon* spp. spillover

Different factors were noted which could lead to the spillover of *Hepatozoon* spp. from wild to domestic hosts via ticks. For instance, locals were found handling, poaching, and hunting of wild animals using dogs. Moreover, dogs were found freely wandering between the habitats of wild and domestic animals, and occasionally feeding on dead wild canids. The overlapped area between domestic and wild animals was found to be increasing as a result of expansion of human settlements and deforestation. Deforestation, which is associated with climate change, was observed to create space for agriculture land and pastures for animal grazing. Under these circumstances, alongside an increase in the population of small generalist hosts like rodents, there was a noticeable decline in overall biodiversity. The invasion of the territory of domestic animals by wild animals were noted in the studied area. Furthermore, the habitat, food sources, and climate were found suitable for both wild and domestic animals, as well as ticks. Using a questionnaire, several other risk factors were gathered (Table 2). Herein, with statistical significance (*P* < 0.05), free ranging animals were at greater risk compared to those confined in shelters (RR = 3.05), animals getting food from wildlife habitat were at greater risk compared to those getting domestic food (RR = 3.06), and animals living in farm buildings located in wildlife habitats were at greater risk compared to those living in farm buildings located in villages (RR = 3.28).

**Table 2:** Details on coupling questionnaire-based risk factors with molecular survey for potential spillover of *Hepatozoon* spp.

**Variable associated with hosts**	**Condition**	**No. of ticks subjected to PCR**	**No. of infected ticks**	**Mean (standard deviation)**	**Relative risk (95% confidence interval)**	***P*-value (χ^2^)**
Gender	Female	117	10	58.5 (68.59)	0.78 (0.30–2.05)	0.62 (0.25)
	Male	55	6	27.5 (30.41)		
Age	3 ≤	46	5	23 (25.46)	1.25 (0.46–3.39)	0.67 (0.18)
	3>	126	11	63 (73.54)		
Farming type	Free-ranging	51	9	25.5 (23.33)	3.05 (1.20–7.54)	0.01 (5.98)
	Confined	121	7	60.5 (75.66)		
Food supply	Wildlife habitat^*^	72	11	36 (35.36)	3.06 (1.11–8.41)	0.02 (5.24)
	Domesticated^•^	100	5	50 (63.64)		
Farm building location	Wildlife zone	58	10	29 (26.87)	3.28 (1.25–8.56)	0.01 (6.54)
	Village	114	6	57 (72.12)		
Farm building nature	Mud	95	8	47.5 (55.86)	0.81 (0.32–2.06)	0.66 (0.20)
	Concrete	77	8	38.5 (43.13)		
Living status	Herd	105	9	52.5 (61.52)	0.82 (0.32–2.10)	0.68 (0.17)
	Single	67	7	33.5 (37.48)		
Altitude	Hilly	56	6	28 (31.11)	1.24 (0.48–3.25)	0.66 (0.20)
	Plain	116	10	58 (67.88)		

^*^Encompassed both free ranging and those confined animals which were provided with fresh forage from wildlife habitat.

^•^Included chaff and straw, cooked and raw food, hay, and mustard cake.

## Discussion

Despite potential agents of spillover of *Hepatozoon* spp. from wildlife to domestic animals, ticks have been widely overlooked as vectors of *Hepatozoon* spp., and there is a scarcity of knowledge regarding *Hepatozoon* spp. of wildlife origin in ticks infesting domestic animals. However, it is crucial to address this knowledge as these parasites pose a potential zoonotic threat, which, in turn could negatively impact food security within ecosystem (38). In addition to highlighting the possible spillover of *Hepatozoon* spp., to the best of our knowledge, it is the first report on the occurrence of *H. canis* in *R. sanguineus* in Pakistan, *H. ayorgbor* in *R. haemaphysaloides* and *H. colubri* in *Ha. sulcata* and *Hy. anatolicum*.

Having a large number of small ruminants, the northern part of Pakistan is considered endemic to *Haemaphysalis* ticks compared to *Hyalomma* and *Rhipicephalus* ticks (39–48). *Haemaphysalis* ticks being the most abundant in this study could be attributed to two main factors. Firstly, a larger portion of the study focused on the north-western KP, and secondly, the inclusion of its two common domestic hosts, goats and sheep. This study revealed that dogs exhibited the highest prevalence of tick infestation, highlighting their status as one of the most overlooked hosts regarding tick control practices. Examined dogs were in close interaction with other domestic animals, including cows, goats, and sheep for their role in protecting animals from predators. Furthermore, knowing the scarcity of DNA based information of *Hepatozoon* and the effectiveness of 18S rRNA in their accurate detection and differentiation (26, 27, 49), the collected ticks in the current endeavor were subjected to PCR targeting 18S rRNA of *Hepatozoon*. On comparison of the obtained sequences, like (26, 50), the differences were considerable to distinguish them as three distinct *Hepatozoon* spp. By clustering of query sequences of the *Hepatozoon* spp. with their corresponding homologous sequences, the phylogenetic analysis confirmed the evolutionary relationship suggested by previous reports (26, 27, 49, 51).

Looking at the details of the vertebrate hosts of the corresponding three *Hepatozoon* spp.; *H. colubri* in snakes (*Zamenis lineatus* and *Zamenis longissimus*), *H. ayorgbor* in snakes (*Python regius*) and the closely related *Hepatozoon* spp. in rats and gerbils (*Rattus exulans, Rattus rattus*, and *Rhombomys opimus*), and *H. canis* in jackals (*Canis aureus*) and dogs (*Canis lupus familiaris*) (20, 52–57), their wildlife origin is prominent. With *H. ayorgbor* detection for the first time in *R. haemaphysaloides* in the present study, previous studies have molecularly detected *H. ayorgbor*-like sequences in ticks, including *Amblyomma rotundatum* infesting snakes (*Chironius multiventris, Corallus hortulanus, Oxyrhopus melanogenys*, and *Philodryas viridissima*) in Brazil, and *Amblyomma fimbriatum* infesting monitor lizard (*Varanus panoptes*) and snake (*Liasis fuscus*) in Australia (58, 59), *Ixodes tasmani* infesting Tasmanian devils (*Sarcophilus harrisii*) in Australia and *Ha. sulcata* (host-seeking) in Turkey (60, 61). Some sequences in GenBank such as MZ475989 belonging to *H. ayorgbor* have been attributed to unknown tick species. Besides the detection of *H. colubri* for the first time in *Hy. anatolicum* and *Ha. sulcata* in the current study, previously *H. colubri*-like species has been detected in *Amblyomma nitidum* infesting reptiles in Japan (62). Unlike previous studies on the detection of *H. ayorgbor*-like sequences and *H. colubri*-like sequences in ticks infesting wild reptiles, herein, *H. ayorgbor* and *H. colubri* were detected in ticks infesting domestic animals. Such preliminary findings require further investigation because the presence of pathogen DNA in a tick species does not guarantee its role as a biological vector. Like previous studies that described *R. sanguineus* as the main vector of *H. canis* in many regions of the world (1, 19, 20, 63), *H. canis* was detected in *R. sanguineus* infesting domestic dogs. Furthermore, the absence of infection in male ticks compared to females and nymphs could indicate that males may not play a role as reservoirs of these parasites due to the fact that they may not feed on wild animals and they die soon after mating with females. Based on the documented evidence of tick infestation on wildlife in the region (44, 64–66), it could be anticipated that the ticks of the current study have acquired the corresponding three *Hepatozoon* spp. from wildlife somewhere during their lifecycle.

A pathogen spillover could be influenced by several factors associated with the pathogen itself, its different types of hosts and climate changes (67, 68), and human behavior. *Hepatozoon* spp. spillover could be determined by their abilities such as infecting a wide host range (1, 69), viability in the environment, adaptability and vector-borne transmission. An increase in the population of generalist small hosts, such as rodents, which are capable of hosting a broad spectrum of pathogens, may contribute to pathogen spillover by different routes, including ticks (68, 70). Additionally, decline in the diversity and abundance of wildlife, and their close proximity to public and domestic animals, may also amplify the spillover events of tick-borne pathogens (68, 71). The decline in biodiversity could be associated with rapid transformations in land use and land cover, as well as their conflict with human due to fears, attacks on crops and livestock, and financial benefits. The factors associated with domestic animals, including grazing in wildlife habitat (free-ranging), feeding on fresh forage from wildlife habitat, and their staying in farm buildings located in the wildlife habitat were noted as considerable risk factors associated with potential spillover. *Hepatozoon* spp. spillover may be facilitated by domestic dogs moving back and forth between wildlife habitats and human settlement (68), which was noted in the current study. Pakistan is the seventh most vulnerable country to climate change (72), which may influence vector dynamics, including ticks, potentially leading to cross-species transmission of *Hepatozoon* spp. Like previous studies, which provided evidences for the spillover of *Hepatozoon* species, including *Hepatozoon americanum, H. canis*, and *Hepatozoon silvestris* (10–12, 73–76), the current study proposes the spillover of *Hepatozoon* spp. from wild to domestic hosts via ticks. *Hepatozoon* spp. may have pathogenicity in unnatural hosts compared to their natural hosts (14–16), potentially posing a significant threat to domestic animals.

## Conclusion

Besides the scarcity of knowledge on ticks as vectors of *Hepatozoon* spp., there is little information on *Hepatozoon* spp. of wildlife origin in ticks infesting domestic animals. Besides detecting *H. canis* in *R. sanguineus*, this is the earliest report on the detection of *H. ayorgbor* in *R. haemaphysaloides* and *H. colubri* in *Ha. sulcata* and *Hy. anatolicum*. By detecting *Hepatozoon* spp. of wildlife origin in ticks infesting domestic animals, this study proposed a possible spillover of *Hepatozoon* spp. from wild to domestic animals through ticks. The current study could assist in understanding the epidemiological surveillance and adopting preventive measures against tick-borne *Hepatozoon* spp. by minimizing the exposure of domestic animals to key risk factors. Further studies are needed in order to uncover ticks' role in the life cycle of *Hepatozoon* spp.

## Data availability statement

The datasets presented in this study can be found in online repositories. The names of the repository/repositories and accession number(s) can be found in the article/supplementary material.

## Ethics statement

The animal studies were approved by the owners of the animals also gave their oral permission to collect ticks from their animals. The studies were conducted in accordance with the local legislation and institutional requirements. Written informed consent was obtained from the owners for the participation of their animals in this study.

## Author contributions

AAli: Conceptualization, Data curation, Funding acquisition, Investigation, Methodology, Project administration, Resources, Software, Supervision, Writing—original draft, Writing—review and editing. HT: Formal analysis, Investigation, Methodology, Visualization, Writing—original draft, Writing—review and editing. MK: Data curation, Investigation, Methodology, Software, Validation, Visualization, Writing—original draft, Writing—review and editing. MA: Data curation, Funding acquisition, Investigation, Methodology, Project administration, Writing—original draft, Writing—review and editing. AAlo: Data curation, Funding acquisition, Investigation, Methodology, Project administration, Resources, Software, Writing—original draft, Writing—review and editing. HA: Data curation, Software, Writing—original draft, Writing—review and editing. TT: Data curation, Formal analysis, Methodology, Project administration, Software, Writing—review and editing. K-HT: Data curation, Formal analysis, Funding acquisition, Methodology, Resources, Software, Visualization, Writing—original draft, Writing—review and editing.
